# Supplementary material for: Patterns of Intron Gain and Loss in Fungi
Source: PLoS Biol. 2004 Nov 30;2(12):e422. doi: 10.1371/journal.pbio.0020422 (PMC532390; doi:10.1371/journal.pbio.0020422)
Supplement: Table S1 — Also available at http://genes.mit.edu/NielsenEtAl/. (4.3 MB ZIP). [file pbio.0020422.st001.zip › NielsenEtAl/html/486.html]

AN2487.1.NCU06394.1.MG06130.1.FG06388.1


```
 CLUSTAL W (1.82) Multiple Sequence Alignments - Introns Inserted


Sequence 1: NCU06394.1	762 aa
Sequence 2: MG06130.1	539 aa
Sequence 3: FG06388.1	749 aa
Sequence 4: AN2487.1	786 aa
Alignment Length: 808 aa
Number Identitical Residues: 264 aa
Alignment Score (without introns) 15671


MG06130.1 	MTPMVLDDQSSAAENAPQPPLREAWKLLPSPTIYTVKEARFEKYHPPQSDGPERATSQ-G
NCU06394.1	MAPSAVDDTVSR---------REMWKTLPDPVLYPVKEAKFEKYIEPQSDGYERAKAQSG
FG06388.1 	MAPSAIDEPAIK---------APEQKTYPPAQIFPVKETRFENYIEPSKDGRERALKH-S
AN2487.1  	MAITSIQSILPARFSINS---SEQGAAKPPPKIFNVYERPFKGYQPPQPDGYEQSRSR-P
          	*:   ::.      .  .          * . :: * *  *: *  *. ** *::  :  

MG06130.1 	GPVAIVIDNG1SSAVRAGWSTENDPRLIVPPIMAKYRDRKLGKTFSFAGWDCYSDTTAKG
NCU06394.1	GNPAIVIDNG1SWAVRAGWSFENAPRLAVPPIMAKYRDRKLGKTFSFAGQDCYADTSARG
FG06388.1 	ESSAIVIDNG1SSTVRAGWSFESKPRFSIPPIMAKYRDRKLGKTFSFAGSDCYADTTARG
AN2487.1  	DTSAIVIDNG1SHLVKAGWSFDKNPRFILPPVMSRYRDRKLNKACQFIGHDAYVDATTRG
          	   ******* *  *:**** :. **: :**:*::******.*: .* * *.* *::::*

MG06130.1 	HIRNAFEAGTDIVSNWDVMEHVLDYLFIKLGR--------------ERQRGLDRQAHS1-
NCU06394.1	HIRNAFEAGTGIVSNWDVMEHVLDYVFLKLGMNGVDGAIDMPIVMTEAVANLPYSRKS1-
FG06388.1 	HIRSAFEVGTGIVSNWDVMEHVLDYIFLKLGMNDADGAIEVPIVMTEAVANLPYSRKS1-
AN2487.1  	QLRYAFDPGTSVVGNWDVMEGVLDYLFIKLGVDGANGGVDRPIVMTEPIANPNYPRKS~E
          	::* **: **.:*.****** ****:*:*** .. ... . .   :*   .     :* .

MG06130.1 	--------------MTEVLFECYGAPSVAYGIDSLFSYKHNKGSTGLVVSSSYSSTHLIP
NCU06394.1	--------------MTEIIFECYGAPSLTYGIDSLFAYRHNQGKTGIVVSSSHSATHLIP
FG06388.1 	--------------MTEIIFECYGAPSLAYGIDSLFSYRHNKGKTGLVVSSSYTSTHVIP
AN2487.1  	KTACEVKLVLTSIVMNEILFECYSAPSVAYGIDSLFSYRYNRGTDGLIVDSSHTSTHVIP
          	.::.. .   ::  *.*::****.***::*******:*::*:*. *::*.**:::**:**

MG06130.1 	VYNQKPLLSQATRLNWGGWHAAEYLLKLIKLKYR-DFLGNVKMNTSQTEHMLRDFCYVSK
NCU06394.1	VYNQKPLLAQATRLNWGGWHAAEYLLKLIKLKYYYGFPG--KLNSSQAENMVRDFCYVSQ
FG06388.1 	VYNNKALLGQATRLNWGGYHNAEYLLKLLRLKYP-AFTG--KTNVSQAEHMLRDHGYVSQ
AN2487.1  	VLNSKALLSNCSRLNFGGMHASEYLLKLMRLKYP-TFPG--KMTEHQMEDLMHNHCYVSK
          	* *.*.**.:.:***:** * :******::***   * *  * .  * *.::::. ***:

MG06130.1 	DYDEELKTYLDWTGLEDRERIIQYPFTEEVIVQKTEEELAKIAERKKESGRRLQEQAAKM
NCU06394.1	DYDSELANYLEWTGLEDRERIVQYPYTEEVINQKTEEELARIAERKKESGRRLQEQAAKM
FG06388.1 	DYDDELASYLEWKGLEDRDIVMQYPYTEIVIEQKTEEELAKIAERKKESGRRLQQQAAKM
AN2487.1  	DYDRELSGYLDWTGLEDRDHVIQYPFTEHIVPEKTEEELARIAERKKESGRRLQEQAAKM
          	*** **  **:*.*****: ::***:** :: :*******:*************:*****

MG06130.1 	RLDRLVKKEQELEYYKKVQKMLVDQT-KKETKRILDDAEVKDEAHLERLIKELEKTIKKA
NCU06394.1	RLERLMKKEQELEYYKDVQRRISEQTTKKEIKRILDDAEVKDEAALDRMVKELEKSIKRA
FG06388.1 	RLEKLMKKEQDLEYYKNLQKNITDVT-KKELRRQLDSNDIKDENQLEKIIKDLEKAVRKA
AN2487.1  	RLEKLMKKEQELEYYKDLQRGLQSET-KKEKTRILDAEDLKDEAHLERLIRDLERSIKRS
          	**::*:****:*****.:*: : . * ***  * **  ::***  *::::::**::::::

MG06130.1 	RTKDLGGEVEDEAAEEPDFSLLEVPDEELDDAGIKAKRQQRLMKSNHEARARAKAEKEAE
NCU06394.1	RTKDLGGEQEEEQ-EAPDFSLLDVPDDQLDEAGLKQKRQQRLMKSNHDARARAKAEKEAE
FG06388.1 	RTKDVGGDPEEEQ-EQPNFDLLDVPDDQLDDAQIKQKRQQRLLKSNHEARARAKAEKEAE
AN2487.1  	RNKDLGNEEQEEASEEMSFPLLDVPDEELDEAGLKEKRHQRLMKSNVDARQRAKEEKERE
          	*.**:*.: ::* :*  .* **:***::**:* :* **:***:*** :** *** *** *

MG06130.1 	KARIAEEARLDEERRTNNLEEWLADKRRAHQ~EAAARVKEREMLSKDMGNRKTLASVMRM
NCU06394.1	KARIAEEARLDEERRTNDLEGWLEEKRQLRL~AKLAQIKERDRLKADLGNRKSLANQIRM
FG06388.1 	KARVVEEERADTERRENDLENWLEEKRQRRA~ETLQKMKERERLKQDLGNRKSLASQIRM
AN2487.1  	QARKDEELRLDREKRENDPEGWVAERRAQRQ0NLLQRIKERDRMKADLGNRKSLASQMRM
          	:**  ** * * *:* *: * *: ::*  :      ::***: :. *:****:**. :**

MG06130.1 	KSIANLASDTPTGSGTSRKRRRGGDDDDFGANDA--------------------------
NCU06394.1	KNLANLASDTPATTG--RKRRRGGDDDDFGADDADWGVYRSVAIGANKGDSDDEEEAEED
FG06388.1 	KSIANLASDAP-----TKKRRRGGDDDNFGADDDDWGVYRQIAVG----DNSDDEHEEED
AN2487.1  	KTLANLAADGP------KKRRRGGDDDDFGANDEDWGVYRTVATG-----EQSDDDEEED
          	*.:****:* *      :*********:***:* . .     : .     .....  ...

MG06130.1 	------------------------------------------------------------
NCU06394.1	LEASVRALEQDLLEYDKEFTYEHTLEAQNDWTKSLLHAFRYGPRPFDAAAPAETHRLHLN
FG06388.1 	LNSALKSLEEDLLRYDPDFDYEHTHEAQSDWSKSLLHAFARGPRPFDPSSQAELNQIHLN
AN2487.1  	LSGMLDSVERELLEYDPEFTENHTLAAQSDWTKSLIHVFLRGPWPFDPESQREAHQIHLN
          	 ..   : . .    . .   . :  :... :.:       .. . .. :  .      .

MG06130.1 	------------------------------------------------------------
NCU06394.1	VERIRVPEVIFQPSAIAGVDQAGIIEIAGDILNQRLVGIPGLDRDSFLKDIFLTGGNTMF
FG06388.1 	VERIRVPEVVFRP-SIAGVDQSGIVDIAGDILNQRLGNVP--NRDDFLRDVFLTGGNTLF
AN2487.1  	VERIRVPEVVFKP-SIAGIDQAGIVEIAADIVNQRFSNPE--EQARLLRDVFLTGGNTLF
          	 .    ..    . : :. ..:.  . :..  ..   .    .      .   :...:  

MG06130.1 	------------------------------------------------------------
NCU06394.1	QGFDERMRASLMPLLPAGTPLTVRKAKDPILDAWKGAAGWAGSEDWQRAKVTREEYQEKG
FG06388.1 	KNFDDRVRDGLRSLLPADSPLTVRRAEDALLDAWKGAAGWVGTPAWKTAKISREEYQEKG
AN2487.1  	QNFDERFRNDFRACLPLEAQLTVRRASDPILDAWKGAAQWASGSGLAKSSISREEYLEKG
          	.. ..    .  .  .  :  :   :...  .: ..::   .      :. : ..  ...

MG06130.1 	-----~-----------~---------------
NCU06394.1	AEYLK0EHDLGNAFS--~---------------
FG06388.1 	PEYIK0EHDMGNSYA--~---------------
AN2487.1  	SEYLK0IGICTQICICT1GLEQAPEISRFIAQT
          	..  .      .   .: . ..:.. :   :.:
```
